# Insights into the Effects of Ligand Binding on Leucyl-tRNA Synthetase Inhibitors for Tuberculosis: In Silico Analysis and Isothermal Titration Calorimetry Validation

**DOI:** 10.3390/biom14060711

**Published:** 2024-06-16

**Authors:** Zia Ur Rehman, Asim Najmi, Khalid Zoghebi

**Affiliations:** Department of Pharmaceutical Chemistry, Faculty of Pharmacy, Jazan University, P.O. Box 114, Jazan 45142, Saudi Arabia; anajmi@jazanu.edu.sa (A.N.); kzoghebi@jazanu.edu.sa (K.Z.)

**Keywords:** computer-aided drug design, virtual screening, leucyl t-RNA synthetase inhibition, molecular dynamics simulation

## Abstract

Incidences of drug-resistant tuberculosis have become common and are rising at an alarming rate. Aminoacyl t-RNA synthetase has been validated as a newer target against *Mycobacterium tuberculosis*. Leucyl t-RNA synthetase (LeuRS) is ubiquitously found in all organisms and regulates transcription, protein synthesis, mitochondrial RNA cleavage, and proofreading of matured t-RNA. Leucyl t-RNA synthetase promotes growth and development and is the key enzyme needed for biofilm formation in Mycobacterium. Inhibition of this enzyme could restrict the growth and development of the mycobacterial population. A database consisting of 2734 drug-like molecules was screened against leucyl t-RNA synthetase enzymes through virtual screening. Based on the docking scores and MMGBSA energy values, the top three compounds were selected for molecular dynamics simulation. The druggable nature of the top three hits was confirmed by predicting their pharmacokinetic parameters. The top three hits—compounds **1035** (ZINC000001543916), **1054** (ZINC000001554197), and **2077** (ZINC000008214483)—were evaluated for their binding affinity toward leucyl t-RNA synthetase by an isothermal titration calorimetry study. The inhibitory activity of these compounds was tested against antimycobacterial activity, biofilm formation, and LeuRS gene expression potential. Compound **1054** (Macimorelin) was found to be the most potent molecule, with better antimycobacterial activity, enzyme binding affinity, and significant inhibition of biofilm formation, as well as inhibition of the LeuRS gene expression. Compound **1054**, the top hit compound, has the potential to be used as a lead to develop successful leucyl t-RNA synthetase inhibitors.

## 1. Introduction

**Figure 1:**
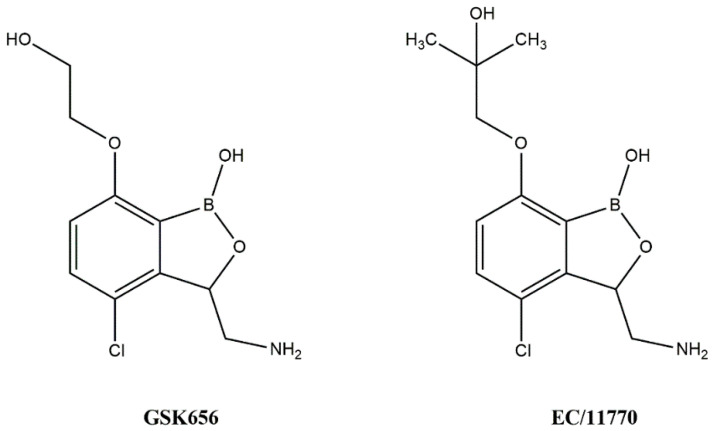
Inhibitors of mycobacterial leucyl t-RNA synthetase.

The WHO Global Tuberculosis Report 2022 estimated that there are about 10.6 million cases of tuberculosis globally, along with 1.6 million deaths [1]. Incidences of drug-resistant tuberculosis have become common and are rising at an alarming rate. The development of resistance and cross-resistance has always driven researchers to search for novel targets and drugs with new mechanisms to counter the drug resistance problem [2,3]. Aminoacyl t-RNA synthetases are ubiquitously found in all organisms and regulate the transcription process by playing an active role in protein biosynthesis. It involves condensation of amino acid with ATP followed by transfer of the aminoacyl group to the 3′ end of homologous t-RNA. Aminoacyl t-RNA synthetases also regulate the mitochondrial RNA cleavage and proofreading of matured t-RNA [4]. High selectivity against bacteria can be achieved because of notable structural differences observed between prokaryotic and eukaryotic aminoacyl t-RNA synthetases in their catalytic domains [5]. Further, the aminoacyl t-RNA synthetases are not easily mutated, which confers it with a lower chance of developing drug resistance [4]. As per the active site topology features, aminoacyl t-RNA synthetases are divided into two types. The first class of enzymes contains a classical Rossmann dinucleotide-binding domain, whereas the second class of enzymes have anti-parallel β-sheets [6]. Leucyl t-RNA synthetase (LeuRS) is one of the first classes of aminoacyl t-RNA synthetase enzymes and has recently been validated as a promising antimycobacterial target. Leucyl t-RNA synthetase contains three different types of domains—an aminoacyl domain, a t-RNA binding domain, and an editing domain (also known as connective peptide) [7]. This editing domain has proofreading action and can hydrolyze any mischarged t-RNAs complexed with wrong aminoacyl groups [8]. Various benzoxaborole derivatives, like AN2690, ZCL039, GSK656, and EC/11770, have been discovered as potent inhibitors, amongst which GSK656 and EC/11770 possess antimycobacterial leucyl t-RNA synthetase [4,8] inhibition properties [9,10]. Some antimycobacterial leucyl t-RNA synthetase inhibitors amongst the N-Benzylidene-thiazol-2-yl-hydrazin [1] and 5-phenylamino-2H-[2,4]triazin-3-one derivatives have also been reported [11,12]. Figure 1 represents the known inhibitors against mycobacterial leucyl t-RNA synthetase.

**Figure 2:**
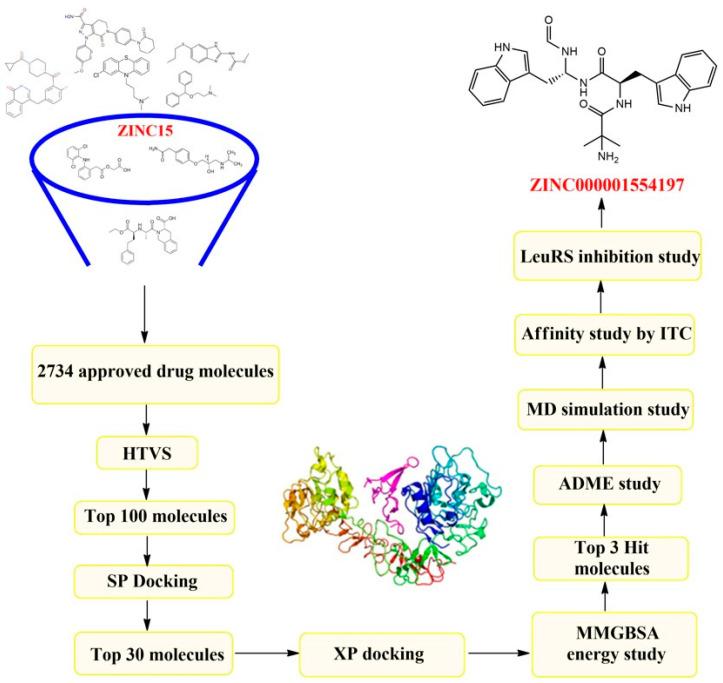
Detailed workflow of the virtual screening.

Researchers are searching for lead molecules against new targets like aminoacyl t-RNA synthetase to prevent the looming threat of drug-resistant tuberculosis. Various computational drug design tools coupled with in silico screening have become an indispensable part of the current drug discovery programs [2]. Molecular simulation studies have shown high efficacy in predicting hit molecules that can be developed into novel drug candidates against leucyl t-RNA synthetase. Virtual screening has been extensively used to screen large databases ranging from small molecules to approved drug molecules to large compounds, both synthetic as well as natural, with diverse chemical structures [13,14]. A database consisting of drug molecules officially approved by various countries’ regulatory authorities was screened. The molecular docking score has been routinely used in structure-based virtual screening to filter the databases. But often, it has been observed that compounds with good docking scores do not reside at the catalytic pocket of the receptor in the aqueous environment of the body. To address this problem, a molecular dynamics simulation study was conducted to find the best candidates for forming stable complexes with the protein. Finally, the top hit candidates were also assessed for their suitable pharmacokinetic and toxicity profiles. In this study, we have employed docking, MMGBSA energy analysis, and dynamics simulation modules of the Schrodinger suite to filter out the top hits against leucyl t-RNA synthetase from the prepared database. A biophysical method and an isothermal titration calorimetry (ITC) were used to study the binding affinity of the hit molecules. Biological activities like inhibition of biofilm and LeuRS gene expression studies were carried out to validate our in silico findings. This drug-repurposing study aims to identify drug molecules that have already been approved by the FDA and various other drug regulatory authorities. The selected drug molecule has the advantages of having known physico-chemical properties, clinical trial data, adverse effects, and safety profiles. Therefore, if these in silico hits are to be selected for clinical trials against tuberculosis, then phase-I and phase-II studies could be skipped as the data are already available. Through structure-based virtual screening, we have identified three in silico hits that were found to have the potential to bind with the leucyl t-RNA synthetase of *Mycobacterium tuberculosis*. To validate the virtual screening results, the in silico hits were subjected to MD simulation, isothermal titration calorimetry, in vitro antimycobacterial activity assays, and tests for biofilm inhibition and LeuRS gene expression. A detailed workflow describing the present virtual screening campaign has been illustrated in Figure 2.

## 2. Materials and Methods

Schrodinger suite (2021-22 release) was used to execute this drug discovery endeavor.

### 2.1. Preparing Protein

The 5AGS.pdb crystal structure of leucyl t-RNA synthetase complexed with 3-(Aminomethyl)-4-bromo-7-ethoxybenzo[c][1,2]oxaborol-1(3H)-ol-adenosine was downloaded from protein data bank. The leucyl t-RNA synthetase crystal structure of *Mycobacterium tuberculosis* was determined by X-ray diffraction method with a resolution of 1.47 Å. Various amino acid residues like Thr336, Leu432, Tyr435, Asp447, Asp450, Met441, and Arg449 are some of the important binding residues that participate in interaction with the co-crystal ligand. This protein was processed, and a grid was generated using “protein preparation wizard” and “receptor grid generation” panel, as per the standard protocol [15,16].

### 2.2. Preparing Database

ZINC15 is an open-source database with thousands of approved drug molecules and drug-like compounds [17]. From this database, 2913 drug molecules were downloaded to prepare an in-house database. All the structures were cleaned up, and the database was screened according to the ring size, number of heteroatoms, and torsional centers, using LigPrep module to yield final 2734 approved drug molecules [18,19].

### 2.3. Structure-Based Virtual Screening

Glide module of Schrodinger suite has 3 modes of docking: HTVS (high-throughput virtual screening), SP (standard precision) docking, and XP (extra precision) docking. HTVS mode of docking with low accuracy was utilized for initial fast screening of the prepared database against 5AGS protein. Based on the HTVS scores, top 100 molecules were put forward for SP docking.

### 2.4. Glide XP Docking

The top 30 molecules obtained from the SP docking were again docked by using extra precision mode of Glide module using standard procedure [20,21,22].

### 2.5. MMGBSA

Prime module of Schrodinger suite was utilized for calculating MMGBSA energy of the docked complex [18].

### 2.6. ADMET Studies

Prior ADMET studies are essential to avoid late-stage failure of drug candidates in clinical trials. QikProp module of the Schrodinger suite was utilized to carry out prediction of pharmacokinetic parameters of top hit compounds [23,24].

### 2.7. Dynamics Simulation Studies

Molecular dynamics simulations for each protein–ligand complex were performed using the Desmond-maestro software package (v5.2) [18,25]. The system for each complex was built up by selecting TIP4P as a solvent model and orthorhombic as a box shape. Buffer method was used to calculate the box size, and the complexes were reoriented to minimize the box volume. Salt and ion placement were excluded within 20 Å. The structure was neutralized by adding sodium as a counter-ion. A default concentration of sodium chloride salt (0.15) was also set to start the system-building process. After system building, the energy minimization was performed using the energy minimization tool in Desmond-maestro at 100 ps. Each minimized system was exported for the production run in the MD simulation tool of the same software package. Further, NPT ensemble class and other default parameters were selected to start the 100 ns molecular dynamics simulation for each system. Obtained results were analyzed using the simulation interaction diagram module of the Desmond Maestro package [26,27].

### 2.8. Isothermal Calorimetry (ITC)

Isothermal titration calorimetry was used to examine the affinity between leucyl t-RNA synthetase (LeuRS) and ligands (compounds **1035**, **1054**, and **2077**). For ITC measurement, LeuRS and ligands (compounds **1035**, **1054**, and **2077**) were procured from Sigma-Aldrich at highest grade available. Isothermal titration calorimetric measurements were conducted with Malvern Instruments (Malvern, UK) at 25 °C and at pH 7.0 (ITC buffer), as described earlier [28]. LeuRS and ligands (compounds **1035**, **1054**, and **2077**) were dissolved in the ITC buffer 5 mM HEPES-KOH, pH 7.0, and the concentration of protein and ligands was determined spectrophotometrically using standard reference extinction coefficients. ITC experiments and measurements for LeuRS with the potential ligands (compounds **1035**, **1054**, and **2077**) were conducted separately, where ligands were allowed to bind with multiple binding sites. In this study, the top three hits (compounds **1035**, **1054**, and **2077**) were subjected to ITC measurements.

### 2.9. Inhibition of Mycobacterial Biofilm Formation

Wild-type *Mycobacterium tuberculosis* was grown in rich LB media overnight. The cell culture was diluted 1:1000 for biofilm inhibition assay. A routine biofilm assay medium was used with added magnesium sulfate, glucose, and casaimino acid that support biofilm formation. Here, 200 µL of wild-type *Mycobacterium tuberculosis* culture + 100 µL biofilm assay medium was added and allowed to be incubated for 8 hr at 37 °C. After incubation, 100 µL crystal violet (0.1% crystal violet) was added to the tube and allowed for 20 min incubation at room temperature. Thereafter, 100 µL acetic acid (25% *v*/*v* acetic acid) was added to each tube and incubated for 15 min at room temperature. The tubes were subjected to absorbance readings at 550 nm. The test compounds 25 µL (10 mM) were added and allowed to be incubated for 30 mins, and absorbance was recorded at 600 nm. A positive control using standard benzoxaborole AN2690 as Leucyl-tRNA [8] synthetase was used [29,30].

### 2.10. Antimycobacterial Activity

Leucyl-tRNA synthetase (LeuRS) inhibition was achieved via compounds **1035**, **1054**, and **2077**, along with the determination of minimum inhibitory concentration. Here, in the study of the MIC determination, *Mycobacterium tuberculosis* strain H37Rv (*ATCC* 25618) was used. The screened ligands’ (compounds **1035**, **1054**, and **2077**) anti-bacterial activity against the ATCC strain, *Mycobacterium tuberculosis* strain H37Rv (*ATCC* 25618), was determined by well diffusion method described previously [31]. The *Mycobacterium tuberculosis* strain H37Rv (*ATCC* 25618) was grown in the Löwenstein–Jensen medium (LJM) as per the recommended growth conditions. The stock solutions of ligands (compounds **1035**, **1054**, and **2077**) were prepared at a concentration of 10 mg/mL in the DMSO solution. Stock solution was further diluted to 1 mL/mL for preliminary screening. For the determination of minimum inhibitory concentration (MIC) of screened ligands (compounds **1035**, **1054**, and **2077**), the ligands were subjected to the serial dilution using DMSO solution at 6.25, 12.5, 25, 50, 100, 200, and 400 µg/mL. *Mycobacterium tuberculosis* strain H37Rv (*ATCC* 25618) was spread on the LJM agar medium. The wells were prepared on LJM agar plate, and 20 µL of each compound in a different concentration was added to the well and allowed to grow for 6 weeks. The zone of inhibition was measured, and MIC was determined.

### 2.11. Leucyl t-RNA Synthetase Gene Expression Inhibition Study

Hypotriploid alveolar basal epithelial cells A459 were used to examine the inhibitory effect of screened compounds on Leucyl-tRNA synthetase mRNA. Hypotriploid alveolar basal epithelial cells A549 were raised in Dulbecco’s modified Eagle’s medium nutrient mixture F-12 Ham (DMEM/F12) at 37 °C in a humidified 5% CO_2_ incubator with supplements of 9% fetal bovine serum, 1% penicillin/streptomycin solution, and 1% L-glutamine. The cells were incubated with screened compounds by adding 25 µL (10 mM) and allowed to rest overnight. Total RNA of overnight grown hypotriploid alveolar basal epithelial cells A459 was isolated. Cells were washed with PBS, and qPCR analyses were carried out in triplicate using a novel fluorescent DNA-binding dye (like SyberGreen I) and an Applied Biosystems 7500 Fast Real-Time PCR System. The primers used here are forward primer (5′-TAATACGACTCACTATAGTCAGGATGGCCGAGCGGTCTA-3′) and reverse primer (5′-TGGTGTCAGGAGTGGGATTCGAACCCAC-3′). The copies of Leucyl-tRNA synthetase gene mRNA were examined per cycle with and without treatment of test compounds. Here, cytosolic Leucyl-tRNA synthetase gene inhibition was examined using test compounds along with a positive control (benzoxaborole AN2690) [9,32,33].

## 3. Results and Discussion

The ZINC15 database was used to prepare an in-house database containing 2913 approved drug molecules by the US FDA and other regulatory authorities across the world. This database was filtered and cleaned up using the LigPrep module to obtain the final 2734 compounds. The crystal structure of leucyl t-RNA synthetase complexed with 3-(Aminomethyl)-4-bromo-7-ethoxybenzo[c][1,2]oxaborol-1(3H)-ol-adenosine was utilized for screening the prepared database. Molecular dynamics simulation, ADMET, and MMGBSA studies were carried out on the top three hits.

### 3.1. Virtual Screening

The in-house database, which obtained 2734 compounds, was initially screened using the HTVS option of the Glide docking module. HTVS is a high-throughput virtual screening that takes less time to screen a large database by reducing the number of conformations and sampling space. The top 100 molecules obtained from the HTVS study were subjected to SP docking, which uses the same algorithm as that of HTVS but with standard precision. Based on the SP docking score of the compounds, the top 30 molecules were selected for further studies. The co-crystallized ligand of the 5AGS protein structure and a standard inhibitor GSK656, which is under clinical trial, has also been included in this virtual screening for a comparative study. The docking scores ranged from −11.172 to −2.556 Kcal/mol, whereas docking scores of the co-crystallized ligand and GSK656 were −6.69 and −6.508 Kcal/mol, respectively. The SP docking scores of the top 30 compounds, along with their structures, are presented in Supplementary Table S1.

### 3.2. Docking and MMGBSA Studies

The top 30 compounds selected from SP docking were further subjected to XP docking and MMGBSA energy analysis. Although XP docking uses a combination of scoring functions with enhanced accuracy and penalty for any violations, it does not take solvation energy and entropy factors into consideration. Therefore, the final selection of the top three hit molecules should be made based on the free energy values of the docked complexes. The prime module of the Schrodinger suite was utilized to determine the accurate free energy binding analysis of the docked complexes.

The MMGBSA energy values ranged from −87.56 to −39.73 Kcal/mol, whereas the standard inhibitor (GSK656) and the co-crystal ligand had MMGBSA values of −69.72 and −54.81 Kcal/mol, respectively. The XP docking scores and MMGBSA energy values of the top 30 molecules are presented in Table 1. All the compounds had a common binding site inside the 5AGS crystal structure of leucyl t-RNA synthetase. Compounds **1035** (ZINC000001543916), **1054** (ZINC000001554197), and **2077** (ZINC000008214483) were selected to be top hit compounds according to their MMGBSA energy values, which are −87.56, −79.79, and −77.34 Kcal/mol, respectively.

### 3.3. Glide XP Docking

**Figure 3:**
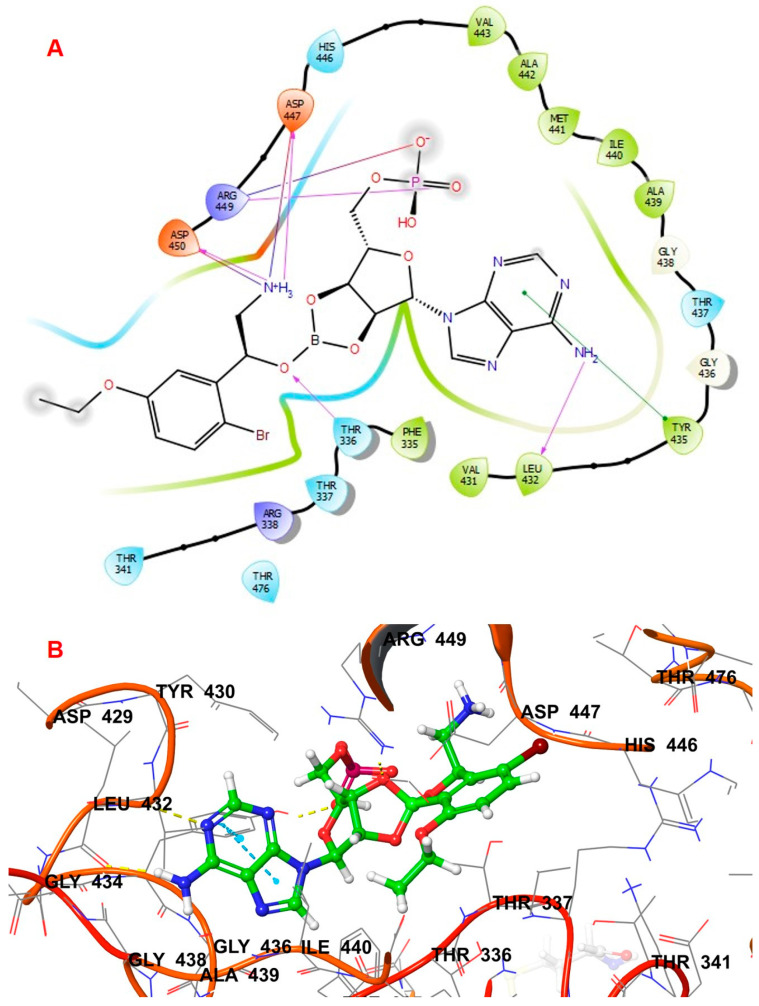
Interactions of the co-crystal ligand inside leucyl t-RNA synthetase. (**A**) 2D pose; (**B**) 3D pose.

Various docking interactions of the top three hit compounds were compared to those of the co-crystallized ligand. The co-crystal ligand of 5AGS was re-docked to the binding cavity of the leucyl t-RNA synthetase. The compound bound to its previous attachment site and achieved an XP docking score of −6.206 Kcal/mol and MMGBSA binding free energy of −54.81 Kcal/mol. The oxaborole and adenosine moiety fitted inside the binding cavity, which was lined with residues like Thr336, Thr337, Leu432, Tyr435, Met441, Asp447, Arg449, and Asp450. The amino group of the adenosine moiety formed a hydrogen bond with the Leu432 residue, whereas the pyrimidine nucleus of the adenosine moiety showed a pi–pi hydrophobic interaction with the Try435 residue. Thr336 residue was involved in hydrogen bonding with the oxygen atom of the methoxy bridge connecting the oxaborole nucleus. Another amino group adjacent to the oxaborole nucleus was also involved in hydrogen bonding with Asp447 and Asp450 residues. The docking interactions of the co-crystal ligand are depicted in Figure 3.

**Figure 4:**
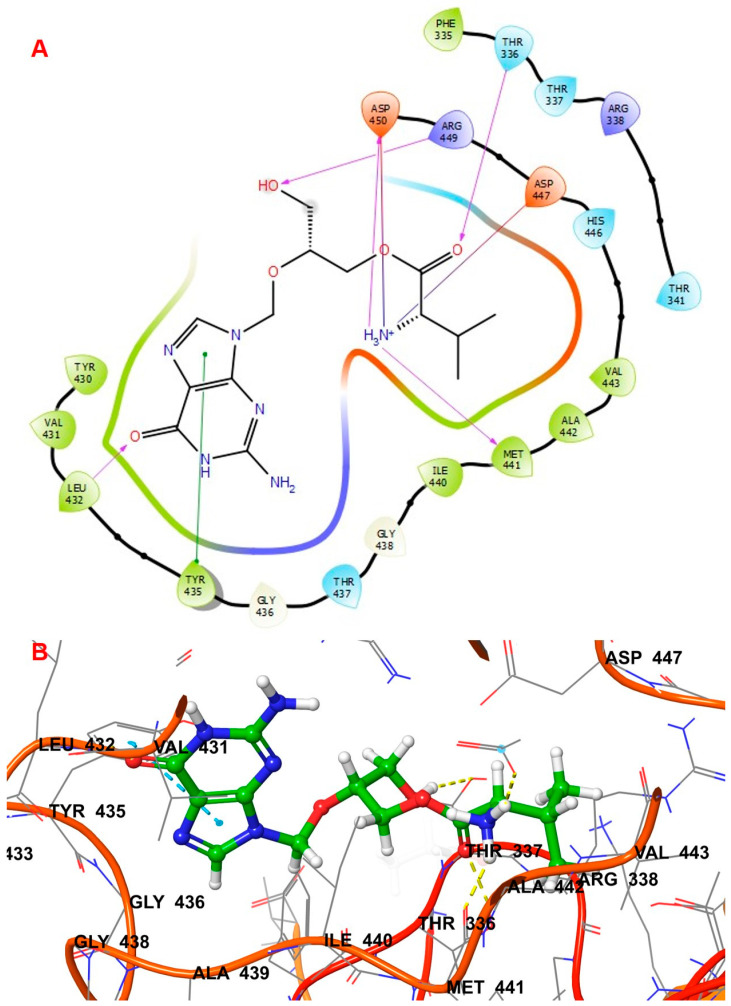
Interactions of 1035 inside leucyl t-RNA synthetase. (**A**) 2D pose; (**B**) 3D pose.

Compound **1035** obtained an XP docking score of −8.819 Kcal/mol and an MMGBSA value of −87.56 Kcal/mol. The purine nucleus was buried inside the binding site, which was stabilized by the pi–pi interaction with the hydrophobic Tyr435 residue. A hydrogen bond was found between the fourth position carbonyl group attached to purine and Leu432 residue. Met441 and Asp450 residues were involved in hydrogen bonds with the amino group attached to the methyl butanoate chain. The ligand was further stabilized inside the binding cavity through hydrogen bonds involving Thr336 with the carbonyl group and Arg449 with the hydroxyl group. The docking interactions of the compound **1035** are depicted in Figure 4.

**Figure 5:**
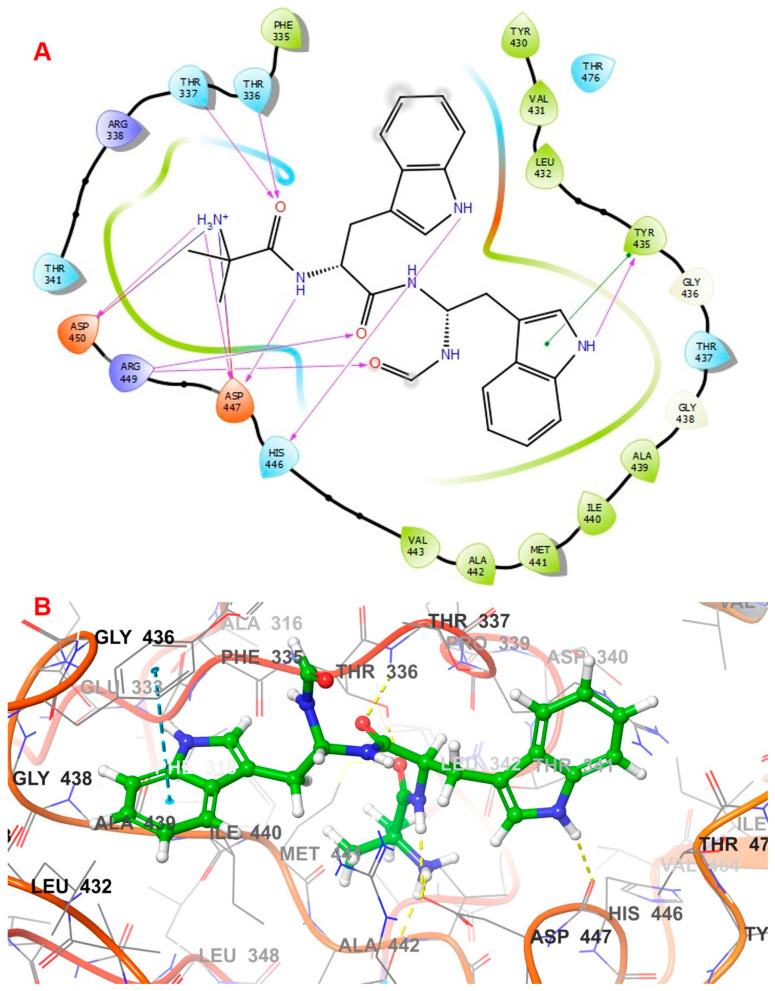
Interactions of 1054 inside leucyl t-RNA synthetase. (**A**) 2D pose; (**B**) 3D pose.

Compound **1054** obtained an XP docking score of −9.006 Kcal/mol and an MMGBSA value of −79.79 Kcal/mol. The indole nucleus attached to the formyl amino branch was tightly fitted inside the binding cavity, stabilized by the pi–pi interaction with the hydrophobic residue Tyr435. The heterocyclic nitrogen atom of the indole moiety also formed a hydrogen bond with Tyr435. Various carbonyl groups were involved in hydrogen bonding with residues Thr336, Thr337, and Arg449. The nitrogen of the indole moiety present at the opposite end formed a hydrogen bond with His446 residue. The terminal amino group was stabilized by hydrogen bonding with Asp447 and Asp450. The amino group attached to the methyl propanamide chain formed a hydrogen bond with residue Asp447. The docking interactions of the compound **1054** are depicted in Figure 5.

**Figure 6:**
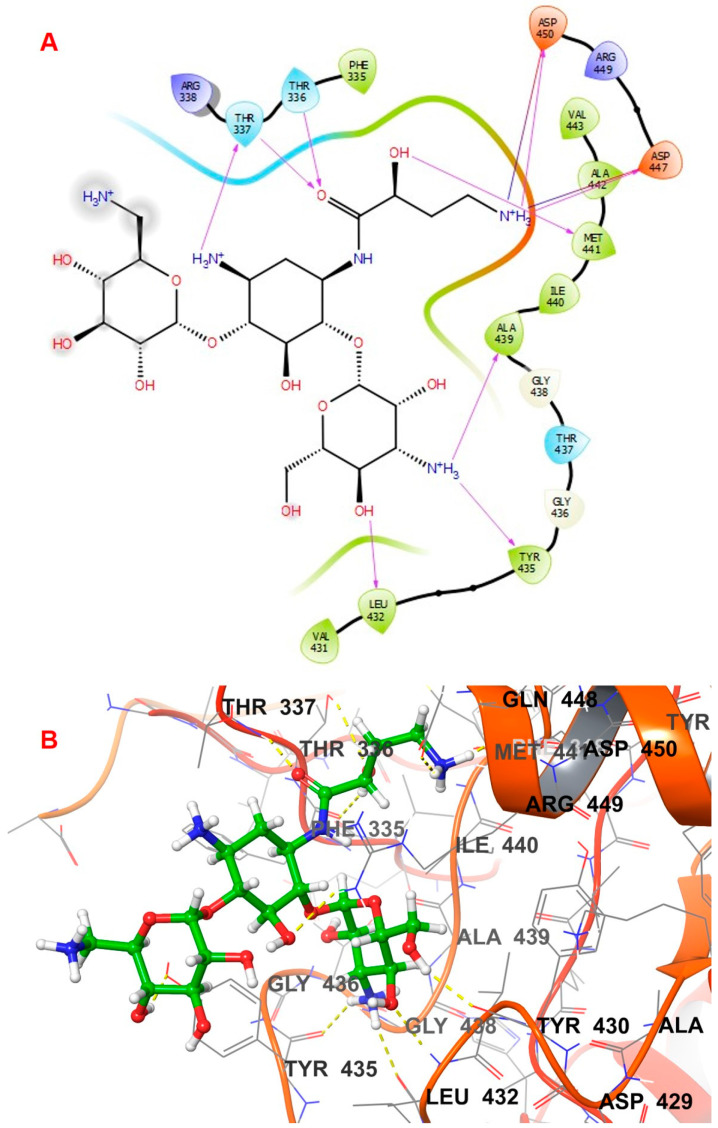
Interactions of 2077 inside leucyl t-RNA synthetase. (**A**) 2D pose; (**B**) 3D pose.

Compound **2077** obtained an XP docking score of −7.986 Kcal/mol and an MMGBSA value of −77.34 Kcal/mol. It is an aminoglycoside where two pyran rings are attached to a cyclohexane through glycosidic linkages. One of the pyran nuclei is stabilized inside the binding site through two hydrogen bonds between Tyr435 and Ala439 with the amino group and another hydrogen bond between Leu432 and the hydroxyl group. The terminal amino group was involved in hydrogen bonds with Asp447 and Asp450 residues. The hydroxyl group attached to the butanamide chain formed a hydrogen bond with residue Met441. The carbonyl group of the butanamide chain also helped in stabilizing the ligand by forming two hydrogen bonds with Thr336 and Thr337 residues. The amino group attached to the cyclohexane skeleton was involved in a hydrogen bond with Thr337 residue. The docking interactions of the compound **2077** are depicted in Figure 6.

### 3.4. MMGBSA Studies

MMGBSA dG Bind is the summation of individual energy contributions due to coulomb energy, covalent binding energy, hydrogen bonding correction, lipophilic energy, pi–pi packing correction, self-contact correction, generalized Born electrostatic solvation energy, and van der Waals energy [34]. Based on the MMGBSA dG Bind values, compounds **1035** (ZINC000001543916), **1054** (ZINC000001554197), and **2077** (ZINC000008214483) were selected as the top three hit molecules. Contribution from MMGBSA dG Bind vdW was found to be maximum, whereas zero contribution was attributed from MMGBSA dG Bind SelfCont. In compound **1035**, the significant contributions for MMGBSA dG Bind were from MMGBSA dG Bind Lipo (−30.54 Kcal/mol), MMGBSA dG Bind Solv GB (33.41 Kcal/mol), and MMGBSA dG Bind vdW (−79.79 Kcal/mol). Similarly, for compound **1054**, important contributions were from MMGBSA dG Bind Lipo (−34.72 Kcal/mol), MMGBSA dG Bind Coulomb (29.14 Kcal/mol), and MMGBSA dG Bind vdW (−69.36 Kcal/mol). Likewise, contributions from MMGBSA dG Bind Lipo (−28.36 Kcal/mol), MMGBSA dG Bind Solv GB (30.81 Kcal/mol), and MMGBSA dG Bind vdW (−66.42 Kcal/mol) were significant for compound **1077**.

### 3.5. Predicting Pharmacokinetic Parameters

A good drug candidate must have favorable pharmacokinetic parameters. As we have screened a database consisting of approved drug molecules, they can be assumed to have suitable pharmacokinetic parameters. QikProp module was used to evaluate the pharmacokinetic parameters of the top three hits, which are presented in Table 2. The molecular weights of compounds **1035** and **1054** were below 500 g/mol, whereas compound **2077** has a molecular weight of 585.607 g/mol. The dipole moment, SASA (total solvent accessible surface area), and volume (total solvent accessible volume) for all three compounds were found to be within the range (i.e., 1 to 12,300 to 100 Å2 and 500 to 2000 Å3, respectively). The van der Waal’s surface area due to polar N, O, and carbonyl C were within the range for compounds **1035** and **1054**, whereas it crossed the upper limit in the case of compound **2077**. Compounds **1035** and **1054** have suitable values for the predicted octanol–water partition coefficient (QPlogPo/w), predicted brain–blood partition coefficient (QPlogBB), predicted skin permeability (QPlogKp), and predicted binding to human serum albumin (QPlogKhsa), but for 2077, the values lie outside the approved range. Except for compound **2077**, the predicted aqueous solubility (QPlogS) values were found to be within the range. All three compounds had poor transport across the gut–blood barrier, as predicted from QPPCaco values. The predicted oral absorption was poor for compounds **1035** and **2077**, but medium absorption was predicted for compound **1054**. The number of violations, as per the rule of five and the rule of three, was within the recommended range for all three compounds.

### 3.6. Dynamics Simulation Studies

Based on the XP docking and MMGBSA energy values, the top three hits were chosen (presented in Table 3) for molecular dynamic (MD) simulation to analyze the structural behavior of the protein–ligand complex. The docked complex stability was assessed by MD simulations for 100ns of the protein–ligand complexes. The changes in the complex conformations were evaluated by the root mean square deviation (RMSD) and root mean square fluctuation (RMSF) values.

**Figure 7:**
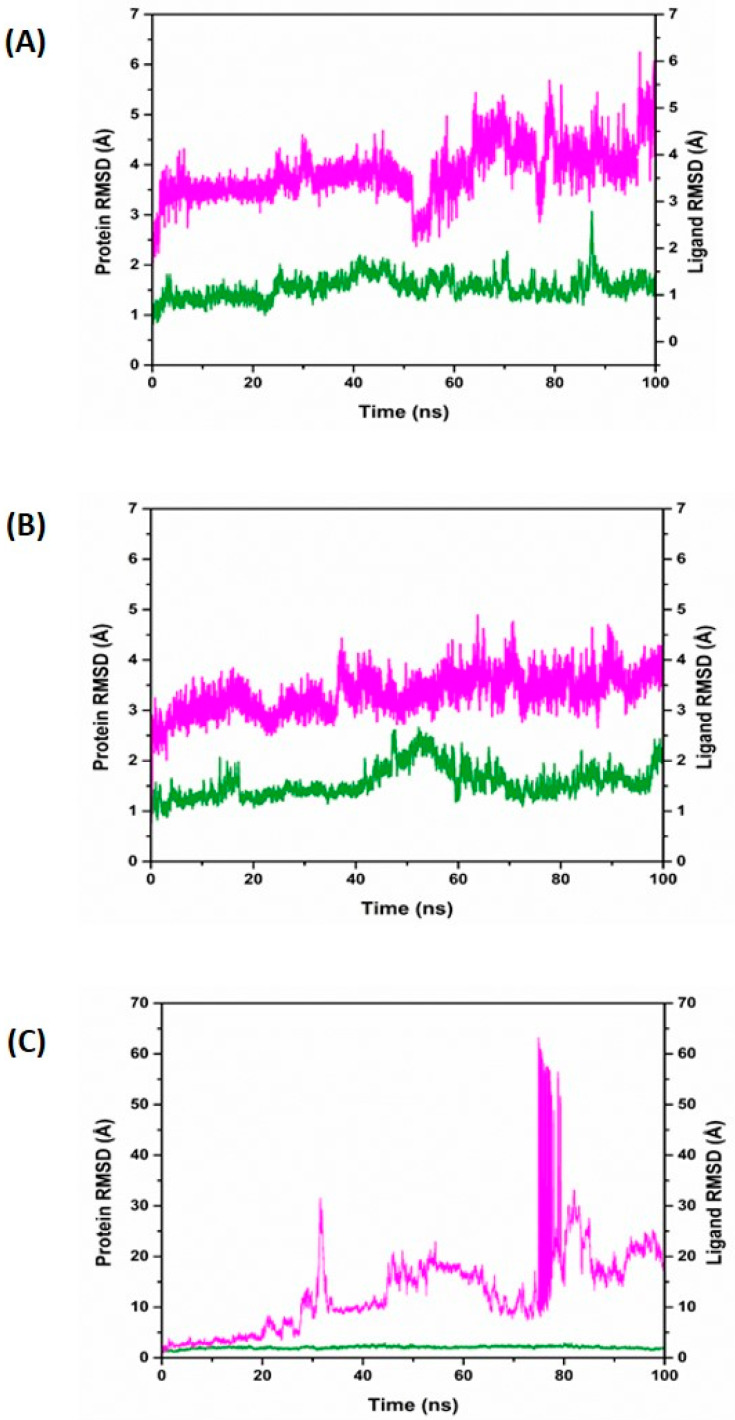
Root mean square deviation (RMSD) of the protein and ligand. Green color represents the target protein, whereas the magenta color represents the compounds, respectively: (**A**) 5AGS_1035 complex; (**B**) 5AGS_1054 complex; (**C**) 5AGS_2077 complex.

The RMSD of protein of the 5AGS_1035 complex in the graph (Figure 7A) shows slight deviations with 1–2 Å, and after 85 ns, it has a gradual increase of 3 Å. Meanwhile, the ligand RMSD appears with an initial RMSD value of 2.5 Å, followed by an increase in RMSD to 4.5 Å; a fall at 55–60 ns has been observed up to 2.5 Å, which then deviated up to 6 Å, with a stable conformation that remains in the protein pocket. The RMSD plot of the 5AGS_1054 complex showed that the protein and ligand were stable and remained in the pocket with acceptable fluctuations during the simulation of 100 ns with a protein RMSD value of 1–2 Å and a ligand RMSD of 2–4 Å (Figure 7B). In contrast, the RMSD plot of the 5AGS_2077 complex shows an initial protein RMSD value between 1.2 and 2.8 Å, which shows less conformational variations, but the ligand RMSD started with small deviations until 20 ns, and then it shows an increase in the values to 30 Å at 25 ns, which again increased up to 60 Å between 75 and 85 ns. At the end of the 100 ns, the fluctuation decreased to 20 Å. Higher fluctuations in the RMSD plot suggested that the ligand might have come out of the binding pocket during the simulation. Therefore, it never reached the equilibrium and demonstrated unstable behavior of the ligand in the binding cavity of the protein (Figure 7C).

**Figure 8:**
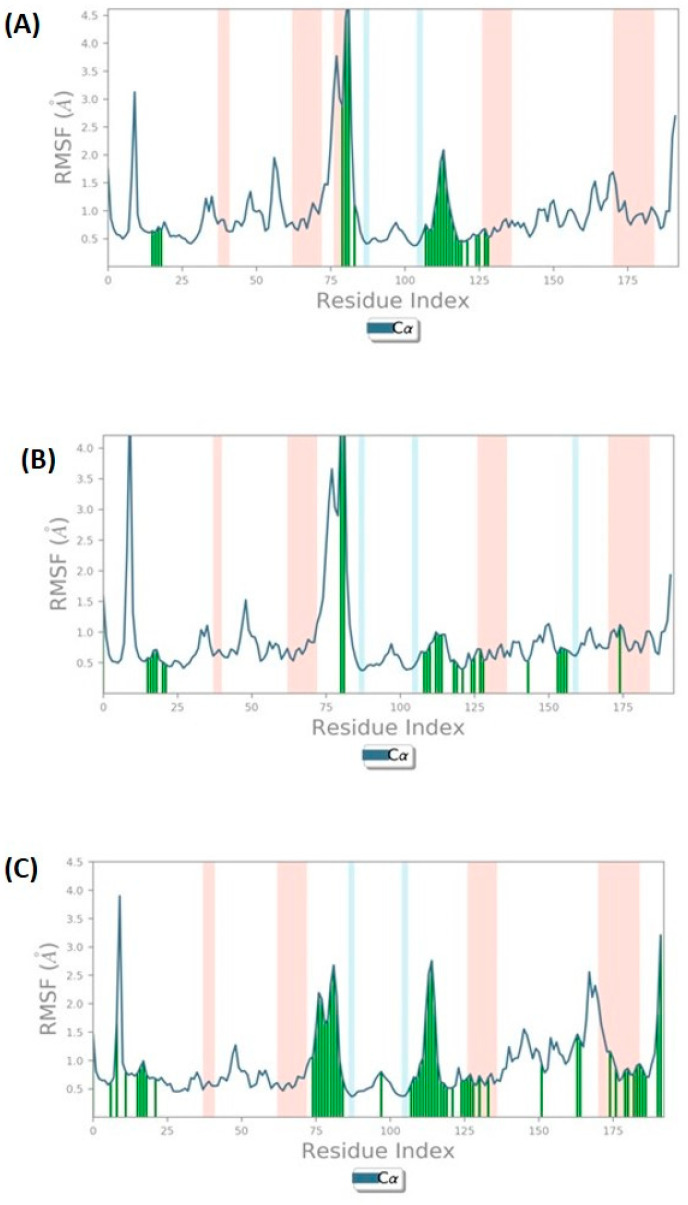
Protein root mean square fluctuation (P-RMSF) of docked complexes. (**A**) 5AGS_1035 complex; (**B**) 5AGS_1054 complex; (**C**) 5AGS_2077 complex. The blue line shows RMSF, the green line represents the interactions formed during simulation, the pink columns indicate alpha helices, and the blue columns indicate beta-strands.

RMSF plots were also evaluated in the molecular dynamics simulation for each residue to determine their flexibility and conformational behavior. The protein RMSF values of these docked complexes were analyzed throughout the 100 ns dynamics simulation, which found that the 5AGS_1035 complex has fluctuation in the Cα of protein with ~4.5 Å (Figure 8A), while the 5AGS_1054 complex experienced this fluctuation until 4 Å (Figure 8B), and 5AGS_2077 complex showed fluctuations up to 3 Å (Figure 8C).

**Figure 9:**
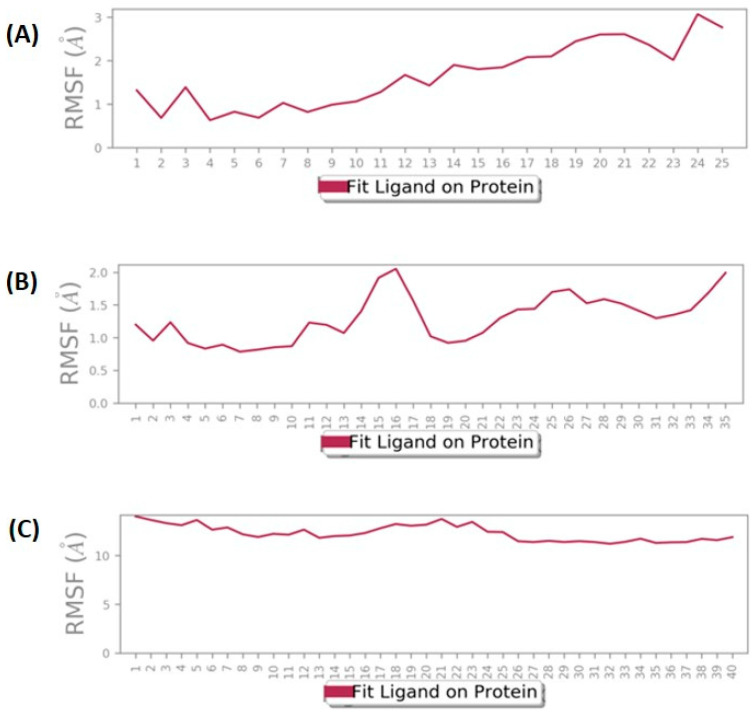
Ligand root mean square fluctuation (L-RMSF) of docked complexes. (**A**) 5AGS_1035 complex; (**B**) 5AGS_1054 complex; (**C**) 5AGS_2077 complex.

The ligand RMSF values showed fluctuations within 1–3 Å in compound **1035** (Figure 9A), with more fluctuations in the C-terminal atoms. Similarly, compound **1054** showed fluctuation in atoms throughout the simulation ranging from 0.5–2 Å (Figure 9B), whereas a higher fluctuation range (above 10 Å) is observed in the case of 5AGS_2077 complex (Figure 9C).

**Figure 10:**
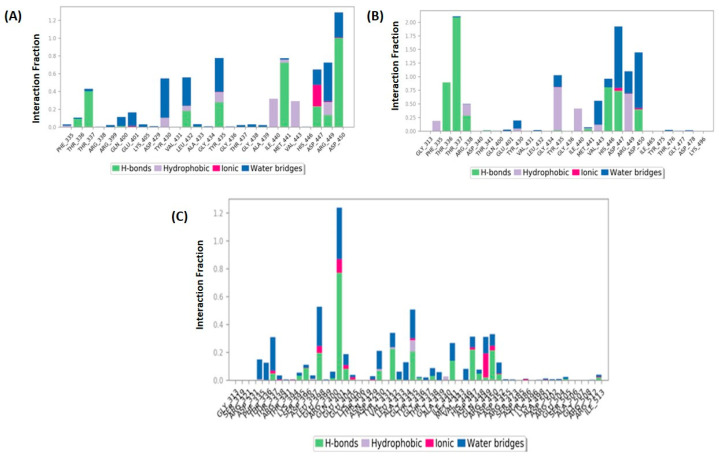
Protein–ligand contact mapping. (**A**) 5AGS_1035 complex; (**B**) 5AGS_1054 complex; (**C**) 5AGS_2077 complex.

**Figure 11:**
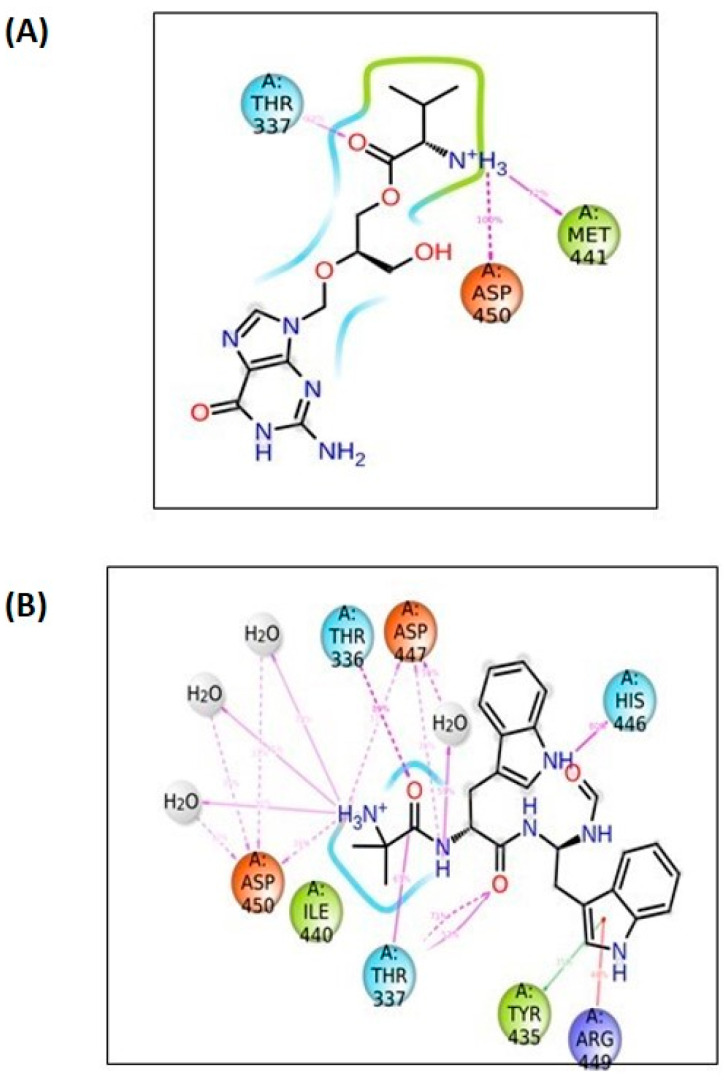
Two-dimensional representation of ligand–protein contacts. (**A**) 5AGS_1035 complex; (**B**) 5AGS_1054 complex.

A protein–ligand mapping plot of the 5AGS_1035 complex (Figure 10A) revealed thatThr337, Leu432, Tyr435, Met441, Asp447, and Asp450 exhibited hydrogen bonding throughout the 100 ns simulation, while some of the residues also showed hydrophobic interactions (Ile440, Val443, Arg449), an ionic interaction in Asp447, and water bridges in some residues. However, the major residues in the ligand–protein contact reveal 39% and 72% contact over the span of the simulation in the Thr337 and Met441 residues, respectively, whereas the Asp450 residue exhibits more than 100% of the hydrogen bond interaction over the span of the entire simulation. The 2D interaction diagram (Figure 11A) revealed that the amino group (attached to the methyl butanoate chain) was involved in hydrogen bonding with Met441 and Asp450 residues. Another hydrogen bond was observed between the carbonyl group (of methyl butanoate chain) and Thr337 residue.

In the 5AGS_1054 complex, the hydrogen bond interactions involved residues like Thr336, Thr337, Arg338, His446, Asp447, and Asp450, whereas hydrophobic interactions involved Tyr435 and Arg449 residues with Thr337 residue accounting for more than 100% of the interaction time. Ligand protein contact (Figure 10B) indicates that Thr337 residue accounted for more than 100% of the interaction time; Tyr435 and Arg449 accounted for 35% and 46%, respectively, during the simulation. The interaction rates for other important residues like His446, Asp447, Asp450, and Thr336 were 80%, 59%, 39%, and 89%, respectively. The 2D interaction diagram (Figure 11B) showed hydrogen bonding with residues Thr336, Thr337, His446, Asp447, and Asp450; pi–pi contacts with Tyr435 and Arg449 residues; and water bridge interactions with Asp447 and Asp450 residues, respectively. The nitrogen of the indole nucleus was involved in a hydrogen bond with His446 residue. The terminal amino group was stabilized by hydrogen bonding with Asp447 and Asp450. The amino group attached to the methyl propanamide chain also formed a hydrogen bond with residue Asp447. The carbonyl groups of the methyl propenamide chain were involved in hydrogen bonding with Thr336 and Thr337 residues. The indole nucleus had shown pi–pi stacking interactions with Tyr435 and Arg449 residues.

In the 5AGS_2077 complex, most of the important residues showed less than 60% hydrogen bond interactions except for Glu401, which accounted for over 70% of the interactions (Figure 10C). No residues were stable in the ligand–protein contact, resulting in an unstable conformational shift in the 5AGS_2077 complex. Even the 2D interaction diagram did not generate any significant interactions. These results correlated well with the higher fluctuations observed in the RMSD plot of the 5AGS_2077 complex. It also confirms that the ligand moved away from the binding pocket during MD simulation and, therefore, lost most of the contacts. These results established the unstable nature of ligand 2077 inside the binding cavity of the protein.

### 3.7. Comparison of Interactions after XP Docking and Dynamics Simulation

The last pose of the MD simulation trajectory was compared with the XP docking results (presented in Table 4) to analyze the interactions that were retained in the hydrophilic environment during simulation.

In compound **1035**, hydrogen bonds with Met441 and Asp450 residues were retained after dynamics simulation, whereas a new hydrogen bond with Thr337 was formed. No other pi–pi stacking and salt bridge interactions were retained during the dynamics study. In compound **1054**, hydrogen bonding interaction with residues Thr336, Thr337, His446, Asp447, and Asp450 were retained during dynamics simulation. Pi–pi stacking with Tyr435 residue was preserved, whereas a new pi–pi interaction with Arg449 residue was also produced. The salt bridge interactions observed in XP docking were retained in the form of water bridges through Asp447 and Asp450 residues. However, compound **2077** could not retain any of the interactions observed during XP docking.

On careful study of the docking, MMGBSA, and molecular dynamics results, compound **1054** or ZINC000001554197 (Macimorelin) was selected as the top hit compound. In the dynamics simulation, Macimorelin showed the maximum number of interactions with better stability (as evidenced by its RMSD and RMSF analysis) than the other two compounds. Macimorelin has two indole rings attached to each other by alkyl amidic linkage. The methyl propenamide substituent helped stabilize the compound inside the binding cavity through the formation of hydrogen bonds with water bridges. The indole nucleus was also helpful in providing pi–pi stacking interaction and involved with a hydrogen bond. Thus, the molecular dynamics study validated the initial results obtained from XP docking and MMGBSA studies.

### 3.8. Isothermal Calorimetry (ITC)

**Figure 12:**
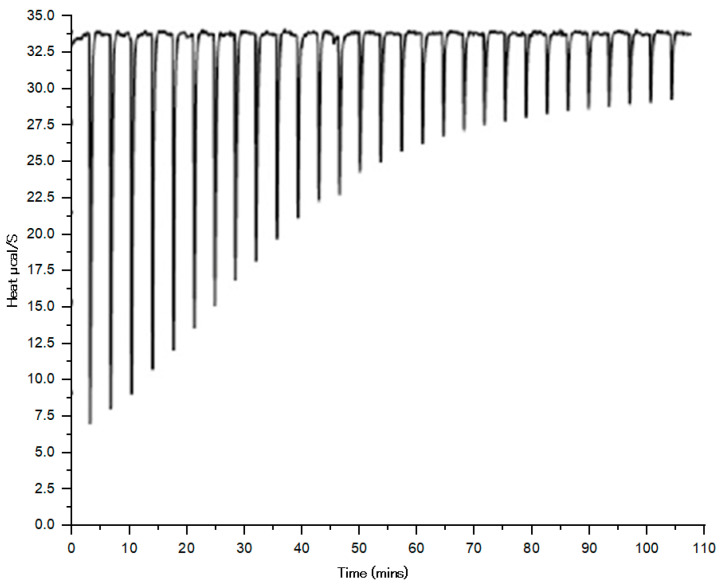
Course of reaction between the ligands and protein LeuRS.

The top three hit molecules were tested for their binding affinity toward the LeuRS protein. Figure 12 demonstrates the course of reaction between the ligands and LeuRS protein until it reaches saturation. After about 105 min, active sites of the protein became completely saturated or occupied with the ligands, as observed by the absence of change in the peaks. As all three ligands have similar binding patterns, they also exhibited quite similar affinity. The results were fitted in the model, and binding parameters, i.e., the affinity between LeuRS and ligands (compounds **1035**, **1054**, and **2077**), were measured via the floating association constant (ka), binding enthalpy (ΔH), and the number of binding sites (n).

**Figure 13:**
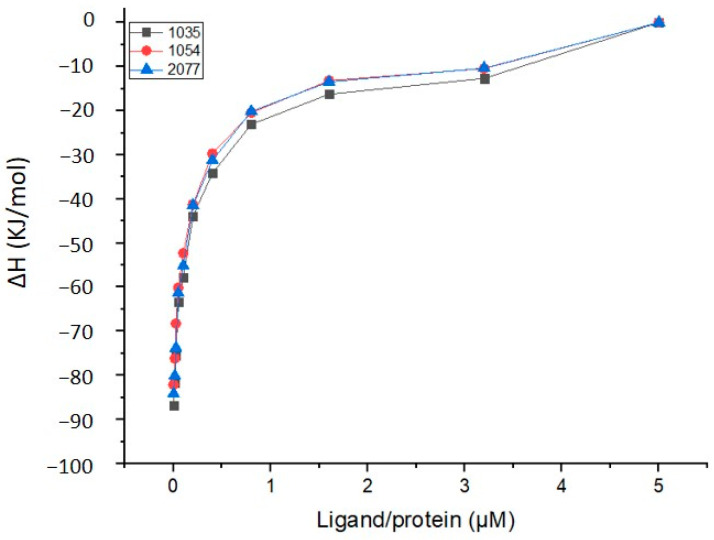
Binding affinity between LeuRS protein and compounds **1035**, **1054**, and **2077**.

The isothermal titration calorimetry measurement of the binding constant between LeuRS and the top three hits at 25 °C temperature and pH 7.0 were summarized in Table 5 and depicted in Figure 13.

The data collected from ITC measurement clearly demonstrate that the affinity of LeuRS with compound **1054** was much higher than the other two ligands used in the present study, i.e., compounds **1035** and **2077**. The higher affinity of compound **1054** was in accordance with the affinity parameter determined via ITC, i.e., the floating association constant (Ka) was reported to be 6.32 × 10^6^, binding enthalpy (ΔH) −48.23 kl/mol, and higher binding sites (n), i.e., 1.72. Table 6 summarizes the ITC results showcasing the floating association constant, binding enthalpy, and the number of binding sites. These findings support the in silico study findings used to test the affinity between LeuRS and other ligands. The affinity of compound **1054** or ZINC000001554197 (Macimorelin) was found to be superior over the other two ligands (compounds **1035** and **2077**), which corroborated the in silico findings. The other two ligands (compounds **1035** and **2077**) also showed promising affinity with the LeuRS and could be potential candidates for the drug development process.

### 3.9. Inhibition of Mycobacterial Biofilm Formation

**Figure 14:**
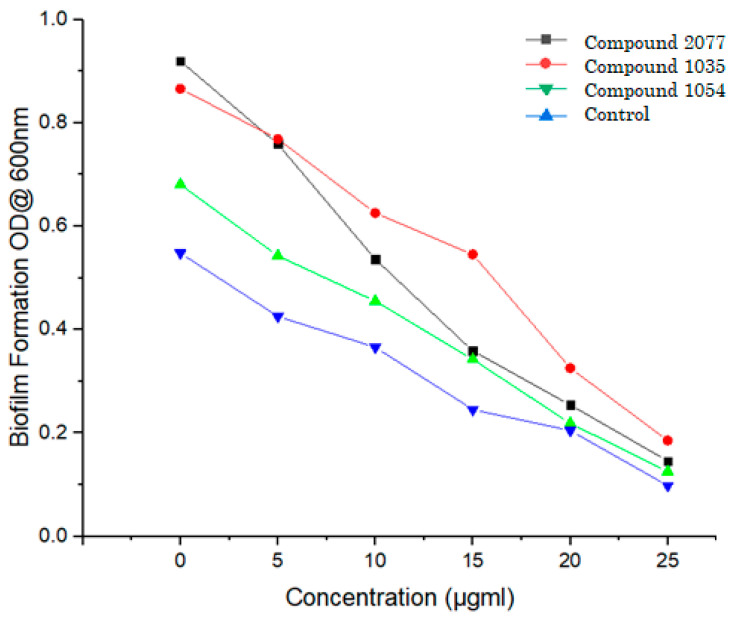
Inhibition of biofilm formation by compounds **1035**, **1054**, and **2077** at different concentrations with control (benzoxaborole AN2690).

In the microbial community, biofilm formation is governed by a series of enzyme-catalyzed extracellular polymeric substances. Leucyl t-RNA synthetase is the key enzyme in biofilm formation in *Mycobacterium tuberculosis*, and it promotes growth and development [29]. Hence, inhibiting the leucyl t-RNA synthetase also affects the biofilm formation and attenuates the *Mycobacterium tuberculosis* growth. Here, in the present study, test compounds (**1035**, **1054**, and **2077**) were examined for biofilm formation inhibitory activity. All three compounds showed promising biofilm formation inhibitory activity as compared to control (data presented in Table 7). Figure 14 demonstrated that compound **1054** had shown higher inhibition in biofilm formation compared to the other two compounds. The findings of this study align with previous research indicating that inhibitors of Leucyl-tRNA synthetase also inhibit biofilm formation by regulating extracellular polymeric substance assembly [32].

Efforts have been continuously made to develop antimycobacterial drugs to combat the growth of *Mycobacterium tuberculosis*. During the drug development process, multiple targets within the MTB proteome have been identified, leading to variations in drug efficacy. In the present study, the antitubercular activity of ligands (compounds **1035**, **1054**, and **2077**) was examined by determining the zone of inhibition. As shown in Table 8, compound **1054** appears more promising compared to 1035 and 2077. The zone of inhibition (ZOI) for 1054 was measured at 18.45 ± 0.19 mm with a minimum inhibitory concentration (MIC) value of 186.39 ± 0.21 µg/mL. Although compounds **1035** and **2077** also exhibited antimycobacterial activity, compound **1054** demonstrated superior efficacy.

### 3.10. Leucyl t-RNA Synthetase Inhibition Study

**Figure 15:**
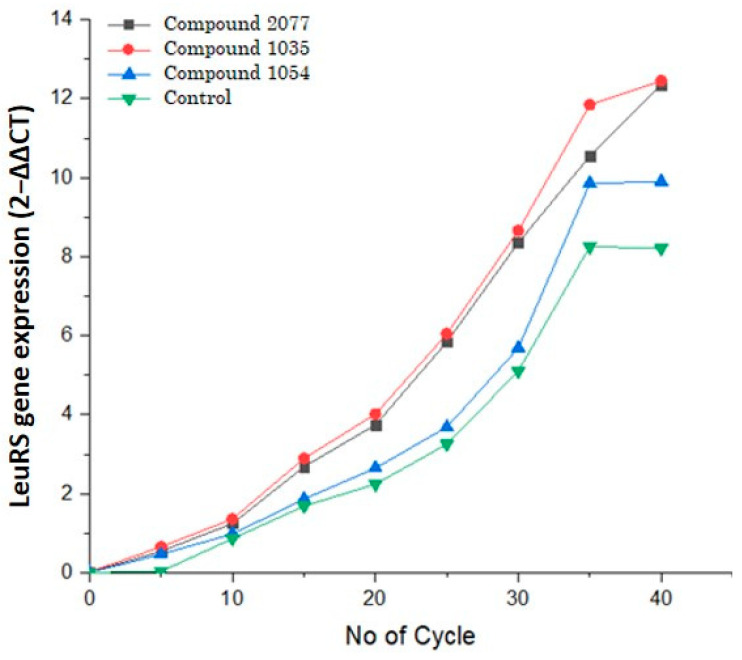
Inhibition of Leucyl-tRNA synthetase (LeuRS) gene expression; copies of mRNA per cycle under the influence of compounds **1035**, **1054**, and **2077**, along with control benzoxaborole AN2690.

Leucyl t-RNA synthetase is the key enzyme responsible for growth, development, and virulence [32]. Therefore, inhibiting this enzyme restricts the survival of the mycobacterial population and the management of tuberculosis. *Mycobacterium tuberculosis* possesses the LeuRS gene in its genome and mitochondria that encode the enzyme. Several studies have demonstrated that cytosolic LeuRS is more functional and effective in the growth and development of *Mycobacterium tuberculosis* [8]. The test compounds, along with the positive control AN2690, were evaluated for translation inhibitory role via inhibiting the LeuRS gene, where mRNA folds per cycle were determined. Certainly, benzoxaborole AN2690 showed lower copies of LeuRS in the RTPCR cycle, whereas compound **1054** was more effective in down-regulating the LeuRS gene as compared to the compounds **1035** and **2077** (Figure 15).

## 4. Conclusions

A generated in-house database with 2734 drug molecules was screened against the leucyl t-RNA synthetase enzyme. Based on the docking scores and MMGBSA energy analysis, the top three hit molecules were selected. These hit molecules had a strong affinity toward the leucyl t-RNA synthetase active site, and their pharmacokinetic parameters were also found to be satisfactory. A molecular dynamics simulation study was carried out to ascertain the best hit molecule in terms of better stability and interactions inside the binding site. Compound **1054** or ZINC000001554197 (Macimorelin) showed comparatively better stability and retained many interactions observed during XP docking. Macimorelin was found to be the most potent among the three hit molecules. A biophysical study by ITC confirmed the superior affinity of compound **1054** toward LeuRS protein. Macimorelin showed significant antimycobacterial and mycobacterial biofilm inhibitory activity along with inhibition of LeuRS gene expression. Therefore, this in silico hit can be used as a lead compound for the development of more leucyl t-RNA synthetase inhibitors to target tuberculosis.

## Figures and Tables

**Table 1:** Top 30 compounds with their XP docking scores and MMGBSA energy values.

Ligand	XP Docking Score	MMGBSA dG Bind
**1035**	−8.819	−87.56
**1054**	−9.006	−79.79
**2077**	−7.986	−77.34
**1425**	−6.908	−75.21
**2350**	−7.584	−74.15
**1098**	−6.394	−73.03
**2102**	−6.326	−73.02
**1008**	−7.245	−70.52
**1256**	−6.001	−67.01
**1104**	−7.974	−61.83
**179**	−7.012	−59.99
**365**	−6.883	−58.99
**207**	−6.864	−58.95
**906**	−7.006	−57.78
**2057**	−5.738	−57.09
**1327**	−6.689	−55.11
**97**	−5.677	−54.91
**2128**	−6.641	−53.97
**2103**	−7.454	−52.49
**2202**	−7.385	−52.22
**479**	−6.294	−52.11
**233**	−6.176	−52.08
**534**	−6.131	−52.01
**1033**	−4.888	−51.05
**2031**	−4.852	−49.67
**194**	−5.582	−48.17
**640**	−4.598	−48.11
**1213**	−5.354	−46.84
**118**	−4.767	−45.71
**2524**	−3.091	−42.38
**1973**	−2.061	−39.73

**Table 2:** Pharmacokinetic parameters.

Ligand	MW	Dipole	SASA	PSA	Volume	QPlog Po/w	QPlogS	QPP Caco	QPlog BB	QPlog Kp	QPlog Khsa	Oral Absorption	Rule of Five	Rule of Three
**1035**	354.365	10.74	623.453	176.713	1098.301	−1.464	−1.054	5.248	−2.457	−7.497	−0.925	1	2	1
**1054**	474.561	6.49	791.223	156.869	1462.906	2.07	−3.236	10.173	−1.824	−5.008	−0.199	2	1	1
**2077**	585.607	11.953	859.859	322.444	1629.256	−8.741	−0.194	0.002	−5.453	−12.732	−2.65	1	3	2

MW: molecular weight; SASA: total solvent accessible surface area; PSA: polar surface area; QPlogPo/w: octanol–water partition coefficient; QPlogS: aqueous solubility; QPPCaco: Caco-2 cell permeability; QPlogBB: brain–blood partition coefficient; QPlogKp: skin permeability; QPlogKhsa: binding to serum albumin protein.

**Table 3:** Top three hit molecules.

Ligand	Structure	IUPAC Name	XP Docking Score	MMGBSA dG Bind
**1035** (ZINC000001543916) Valganciclovir	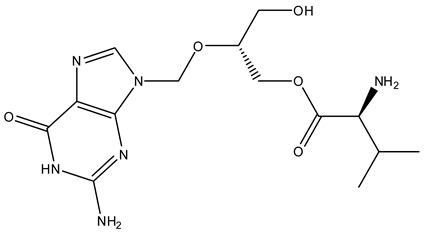	2-((2-amino-6-oxo-1H-purin-9(6H)-yl)methoxy)-3-hydroxypropyl 2-amino-3-methylbutanoate	−8.819	−87.56
**1054** (ZINC000001554197) Macimorelin	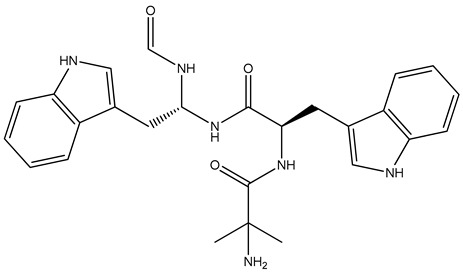	2-amino-N-((R)-1-(((R)-1-formamido-2-(1H-indol-3-yl)ethyl)amino)-3-(1H-indol-3-yl)-1-oxopropan-2-yl)-2-methylpropanamide	−9.006	−79.79
**2077** (ZINC000008214483) Amikacin	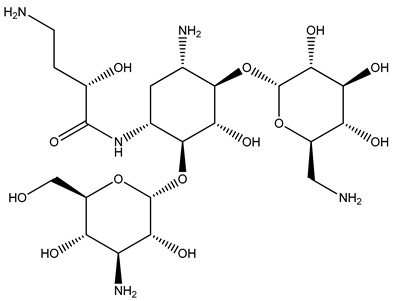	(S)-4-amino-N-((1R,2S,3S,4R,5S)-5-amino-2-(((2S,3R,4S,5S,6R)-4-amino-3,5-dihydroxy-6-(hydroxymethyl)tetrahydro-2H-pyran-2-yl)oxy)-4-(((2R,3R,4S,5S,6R)-6-(aminomethyl)-3,4,5-trihydroxytetrahydro-2H-pyran-2-yl)oxy)-3-hydroxycyclohexyl)-2-hydroxybutanamide	−7.986	−77.34

**Table 4:** Interactions before and after MD simulation.

Compound	Interactions					
	After XP Docking			After MD Simulation		
	H Bonding	Pi–Pi	Salt Bridge	H Bonding	Pi–Pi	Water Bridge
**1035**	Thr336, Leu432, Met441, Arg449, Asp450	Tyr435	Asp447, Asp450	Thr337, Met441, Asp450	-	-
**1054**	Thr336, Thr337, Tyr435, His446, Asp447, Arg449, Asp450	Tyr435	Asp447, Asp450	Thr336, Thr337, His446, Asp447, Asp450	Tyr435, Arg449	Asp447, Asp450
**2077**	Thr336, Thr337, Leu432, Tyr435, Ala439, Met441, Asp447, Asp450	-	Asp447, Asp450	-	-	-

**Table 5:** Constant binding between LeuRS protein and the ligands.

Conc. of Ligand/Protein (µM)	ΔH (kJ/mol) for Ligands		
	1035	1054	2077
00	−86.84	−82.11	−84.16
0.0125	−81.65	−76.15	−80.13
0.025	−75.56	−68.25	−73.79
0.050	−63.41	−60.11	−61.36
0.100	−57.85	−52.27	−55.16
0.200	−43.88	−41.25	−41.42
0.400	−34.16	−29.73	−31.25
0.800	−23.10	−20.46	−20.16
1.600	−16.25	−13.16	−13.45
3.20	−12.65	−10.42	−10.36
5.00	00	00	00

**Table 6:** ITC measurements of compounds **1035**, **1054**, and **2077** against LeuRS.

Ligands	Floating Association Constant ka (1/M)	Binding Enthalpy ΔH (kJ/mol)	Binding Sites (n)
**1035**	4.18 × 10^4^	−49.16	1.14
**1054**	6.32 × 10^6^	−48.23	1.72
**2077**	7.74 × 10^3^	−49.87	0.85

**Table 7:** Biofilm formation inhibitory activity by compounds **1035**, **1054**, and **2077** at different concentrations with control (benzoxaborole AN2690).

2077		1035		1054		Control	
Conc.	OD	Conc.	OD	Conc.	OD	Conc.	OD
0	0.9195	0	0.8658	0	0.6804	0	0.5482
5.0	0.7588	5.0	0.7685	5.0	0.5428	5.0	0.4256
10.0	0.5358	10.0	0.6255	10.0	0.4548	10.0	0.3658
15.0	0.3585	15.0	0.5454	15.0	0.3425	15.0	0.2452
20.0	0.2544	20.0	0.3256	20.0	0.2186	20.0	0.2048
25.0	0.1456	25.0	0.1854	25.0	0.1254	25.0	0.0980

**Table 8:** Antimycobacterial activity of compounds **1035**, **1054**, and **2077** and minimum inhibitory concentration determination.

Ligands	Anti-Mycobacteial Activity	
	Zone of Inhibition (mm)	MIC (µg/mL)
**1035**	13.68 ± 0.21	119.87 ± 0.10
**1054**	18.45 ± 0.19	186.39 ± 0.21
**2077**	15.57 ± 0.80	147.43 ± 0.80

## Data Availability

Data are contained within the article.

## Supplementary Materials

The following supporting information can be downloaded at: https://www.mdpi.com/article/10.3390/biom14060711/s1, Table S1: Structure and docking scores of compounds.
