# Supplementary material for: Insights into the Effects of Ligand Binding on Leucyl-tRNA Synthetase Inhibitors for Tuberculosis: In Silico Analysis and Isothermal Titration Calorimetry Validation
Source: Biomolecules. 2024 Jun 16;14(6):711. doi: 10.3390/biom14060711 (PMC11201714; doi:10.3390/biom14060711)
Supplement: Supplementary file 1 [file biomolecules-14-00711-s001.zip › biomolecules-3009145-supplementary.pdf]

Supplementary Information

In silico guided and in vitro supported drug repurposing strategy for discovery of safe and effective leucyl-t-RNA synthetase inhibitors against tuberculosis

Table S1: Structure and Docking scores of compounds

Ligand	Structure	SP Docking score
1035 (ZINC000001543916)	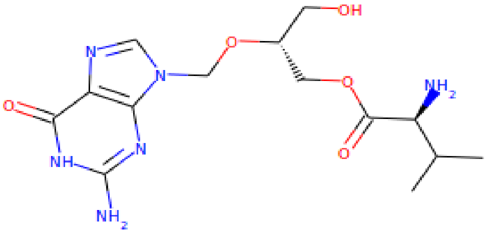	-11.172
2077 (ZINC000008214483)	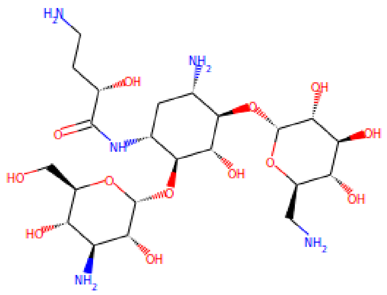	-10.786
1054 (ZINC000001554197)	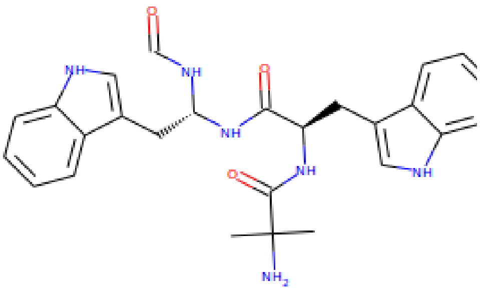	-9.396

1033<br>(ZINC000001543475)	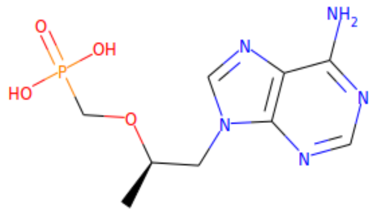	-8.998
906<br>(ZINC000001530713)	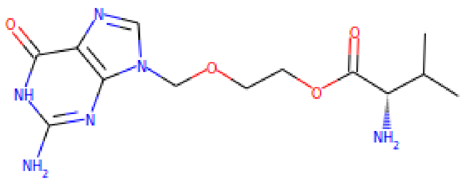	-8.584
2031<br>(ZINC000006507052)	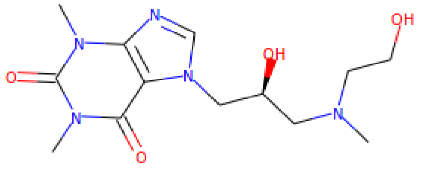	-8.34

194<br><br>(ZINC000000001899)	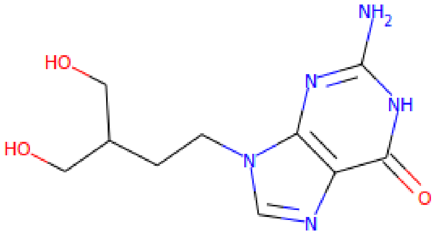	-8.326
640<br><br>(ZINC000000608172)	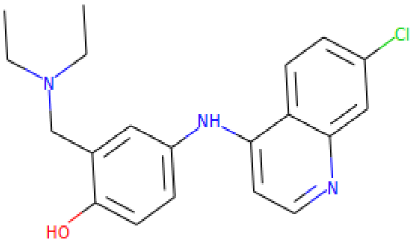	-8.245
97<br><br>(ZINC000000000843)	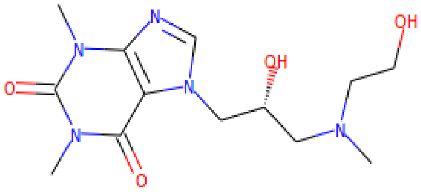	-8.221

2103<br>(ZINC000008215434)	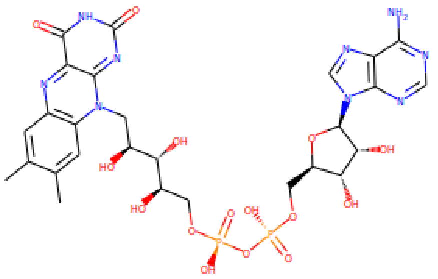	-8.174
2202<br>(ZINC000013507674)	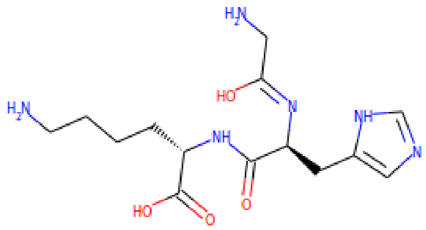	-8.012
479<br>(ZINC000000119895)	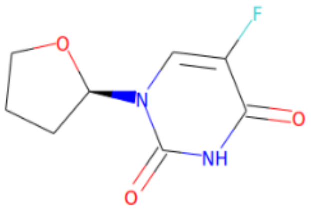	-7.883

233<br><br>(ZINC000000002235)	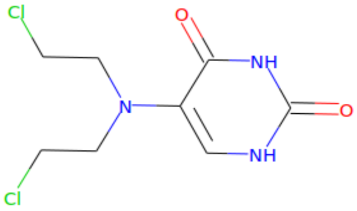	-7.864
2057<br><br>(ZINC000008035395)	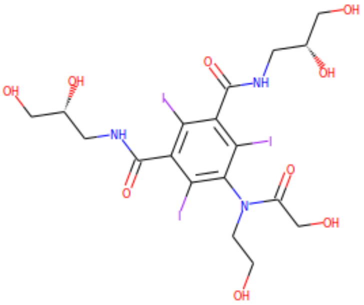	-7.806
1327<br><br>(ZINC000003795098)	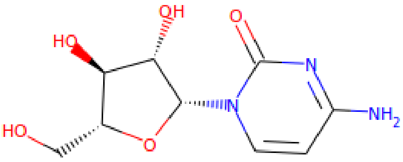	-7.738

2102<br>(ZINC000008215403)	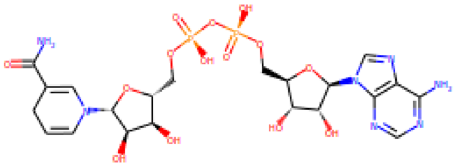	-7.689
534<br>(ZINC000000403567)	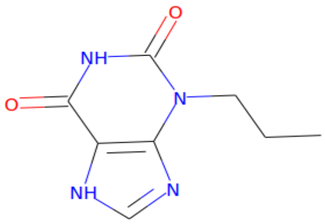	-7.677
1425<br>(ZINC000003821234)	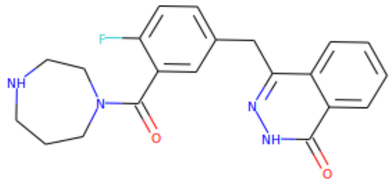	-7.641
179<br>(ZINC000000001695)	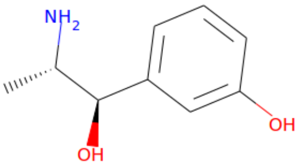	-7.454

365<br>(ZINC000000039092)	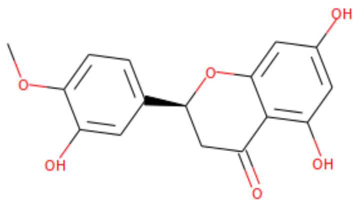	-7.385
Co-crystal ligand	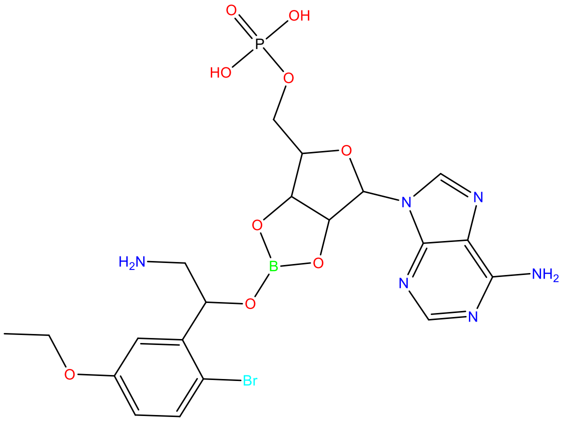	-6.69
GSK656	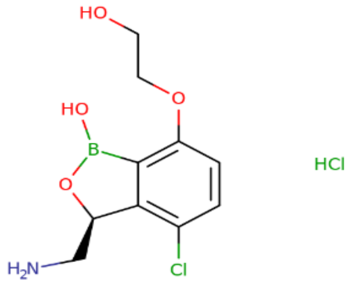	-6.508
207<br>(ZINC000000002028)	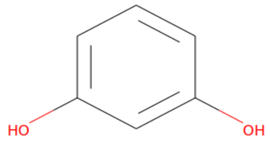	-6.294

1008<br>(ZINC000001535903)	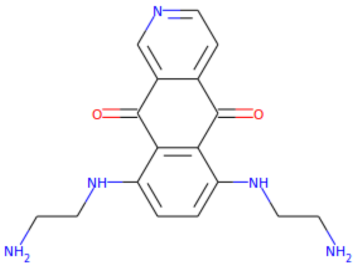	-6.176
1256<br>(ZINC000002584391)	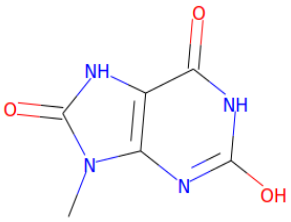	-6.131
1104<br>(ZINC000001843030)	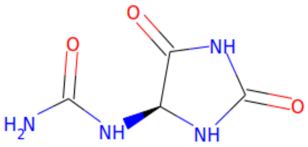	-5.888
2350<br>(ZINC000019632917)	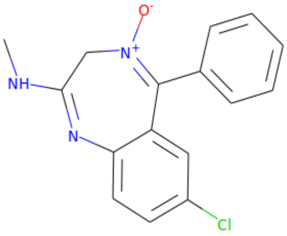	-5.852

1098<br><br>(ZINC000001751811)	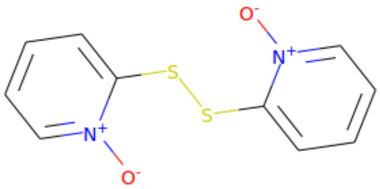	-5.582
1213<br><br>(ZINC000002038451)	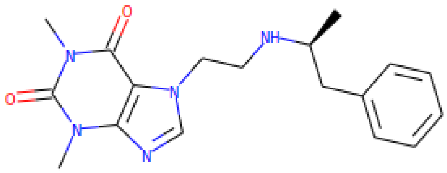	-5.58
118<br><br>(ZINC000000001084)	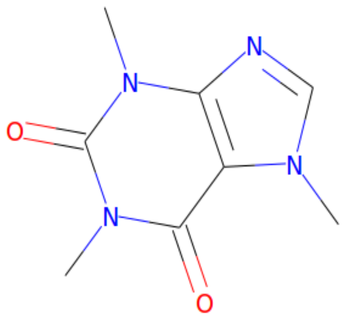	-5.354

2128<br><br>(ZINC000008673078)	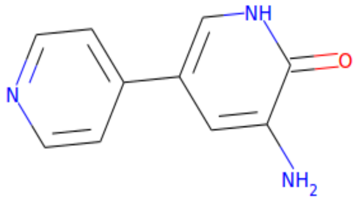	-4.767
2524<br><br>(ZINC000049637509)	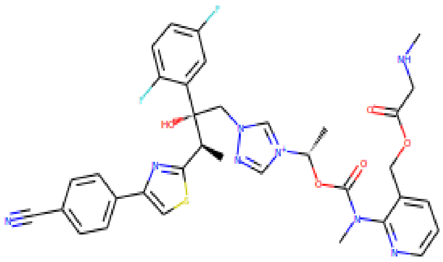	-3.209
1973<br><br>(ZINC000004693575)	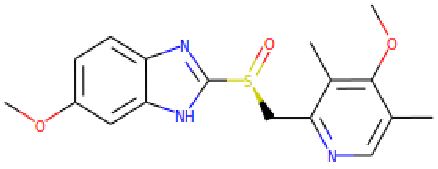	-2.556
